# Impact of a pulsed xenon disinfection system on hospital onset *Clostridioides difficile* infections in 48 hospitals over a 5-year period

**DOI:** 10.1186/s12879-021-06789-y

**Published:** 2021-10-20

**Authors:** Sarah Simmons, Grady Wier, Antonio Pedraza, Mark Stibich

**Affiliations:** Xenex Disinfection Services, 1074 Arion Circle, Suite 116, San Antonio, TX USA

**Keywords:** *C. difficile* infection, Pulsed xenon ultraviolet, Healthcare associated infections, Environmental interventions

## Abstract

**Background:**

The role of the environment in hospital acquired infections is well established. We examined the impact on the infection rate for hospital onset C*lostridioides difficile (HO-CDI)* of an environmental hygiene intervention in 48 hospitals over a 5 year period using a pulsed xenon ultraviolet (PX-UV) disinfection system.

**Methods:**

Utilization data was collected directly from the automated PX-UV system and uploaded in real time to a database. HO-CDI data was provided by each facility. Data was analyzed at the unit level to determine compliance to disinfection protocols. Final data set included 5 years of data aggregated to the facility level, resulting in a dataset of 48 hospitals and a date range of January 2015–December 2019. Negative binomial regression was used with an offset on patient days to convert infection count data and assess HO-CDI rates vs. intervention compliance rate, total successful disinfection cycles, and total rooms disinfected. The K-Nearest Neighbor (KNN) machine learning algorithm was used to compare intervention compliance and total intervention cycles to presence of infection.

**Results:**

All regression models depict a statistically significant inverse association between the intervention and HO-CDI rates. The KNN model predicts the presence of infection (or whether an infection will be present or not) with greater than 98% accuracy when considering both intervention compliance and total intervention cycles.

**Conclusions:**

The findings of this study indicate a strong inverse relationship between the utilization of the pulsed xenon intervention and HO-CDI rates.

## Introduction

The role of the environment in transmission of hospital acquired infections (HAI) is well established [1, 2]. Manual cleaning has been shown to inadequately remove pathogens from the environment [3], and these pathogens may persist on surfaces for months [4]. This inadequacy of manual cleaning can be directly linked to an increased risk of infection acquisition for subsequent patients, with a reported increased risk of 135% for patients in previous *Clostridioides difficile* isolation rooms [5–7]. Hospital-onset *C. difficile* infection (HO-CDI) creates a large burden of disease, causing an estimated 223,900 infections and 12,800 deaths annually [8]. HO-CDIs contribute billions of dollars in direct costs to the US healthcare system annually [9].

Colonized or infected patients are able to heavily contaminate the environment by shedding organisms onto surfaces around them. In rooms of patients with active *C. difficile* infection, gloved hands become just as contaminated when contacting the patient’s environment as when contacting the patient’s skin [10]. Additionally, in a multi-center study assessing the distribution of *C. difficile* spores in the hospital environment, 33% of rooms not currently housing *C. difficile* patients were still culture positive for spores [11]. The authors speculate that the source of this contamination may originate from the previous occupant of the room, or the hands of healthcare workers. A direct observation of healthcare workers found a complex web of interaction between the hands of workers, the patient, and the patient environment [12]. A study using harmless DNA markers as a surrogate for pathogens found that the DNA could be recovered from more than half of sampled surfaces within a neonatal pod after inoculation of only one surface [13]. Additionally, the DNA markers could be recovered from 18% of surfaces in adjacent pods, demonstrating that transmission may not be contained to an individual unit or pod.

Numerous interventions, applied both individually and as bundles, have been implemented in an attempt to interrupt the environmental transmission of HO-CDIs. In the last decade, many hospitals have employed enhanced disinfection with ultraviolet light to combat increasing HO-CDI rates. A multi-center randomized controlled trial assessing a low-pressure mercury vapor UV device showed no change in the HO-CDI rate when the device was utilized for terminal cleaning of *C. difficile* isolation rooms (relative risk 1.00; 95% CI 0.57–1.75) [14].

Pulsed xenon UV devices (PX-UV) have been shown to reduce the environmental bioburden of *C. difficile* in rapid 5 min disinfection cycles [15]. These short cycles allow for more rooms per day to be disinfected, covering more square footage within a healthcare facility. A meta-analysis of studies on the use of PX-UV for enhanced disinfection showed a significant reduction in the rates of HO-CDI acquisition (incidence rate ratio 0.73; 95% CI 0.57–0.94) [16]. In many of the included studies, disinfection with PX-UV was intended for all patient rooms on specific targeted units, not only isolation rooms. When reviewing the studies individually, there appeared to be a relationship between the reported reduction in HO-CDI and to the frequency of use of the PX-UV devices. To further investigate the interaction between increased PX-UV utilization and HO-CDI infection rates, this study performs a retrospective data review of facilities that have implemented PX-UV disinfection programs.

## Methods

### Inclusion/Exclusion criteria

All healthcare facilities located in the United States with a deployed PX-UV disinfection system at the time of analysis were eligible for inclusion in the data set. The National Healthcare Safety Network (NHSN) definition for HO-CDI was changed at the beginning of 2015. Therefore, data collected before 2015 was excluded. At the time of analysis, insufficient data was available for 2020. The final assessed data range was January 2015-December 2019. Data was only analyzed within “target units” of the included facilities, meaning units where a PX-UV device has been deployed with the stated goal of using the device for all discharges and transfers. While other units within the hospital may have had sporadic utilization of the PX-UV device, these were not included in the analysis. For some target units in the data set, there were only partial records for discharges and transfers. Any hospital missing more than 30% of these records from its target units were excluded from the analysis. Predictive mean matching was used to populate remaining missing records. Target units with no HO-CDI during the entire study period were excluded. The final record count was 6,327 before facility-level aggregation from 48 unique hospitals.

### Data collection

Utilization data for target units was uploaded automatically by the PX-UV systems through a real-time cloud-based portal. This data includes values such as unique user name, room number, number of disinfection positions, duration of disinfection cycles and time/date stamps for device use. Data on discharges and transfers, HO-CDI incidence and patient days were provided by the hospitals at the unit level, and were summarized as monthly aggregates. Data is included from a pre-implementation period where there was no utilization of the PX-UV device for most facilities. Six facilities had the baseline period prior to 2015, therefore, this data was excluded for the reasons described previously. The time frame for this pre-implementation data ranges from one month to 36 months, and represents approximately one third of the dataset. This baseline data was included as a quasi-control for variations in *C. difficile* rates prior to implementation of the PX-UV program.

Discharges and transfers were defined as all instances in which a patient room was vacated because a patient was discharged home or to another facility, transferred to another room within the hospital, or declared deceased. All discharges or transfers on target units were considered eligible for PX-UV disinfection. HO-CDI data was defined using the NHSN Lab ID criteria of a positive lab test for *C. difficile* more than 2 days after admission to the facility. Patient days were recorded using a standard once-daily census method.

### Intervention

The pulsed xenon ultraviolet (PX-UV) disinfection system (Xenex Disinfection Services, San Antonio, TX) is an automated, no touch disinfection system that uses broad spectrum (200-280 nm), high intensity ultraviolet light to deactivate pathogens. For each hospital site, target units with higher rates of *C. difficile* transmission were identified and PX-UV systems deployed for additive terminal disinfection. Due to shadows cast by furniture and equipment in the room, UV disinfection devices are most effective when used in multiple positions to maximize direct line of sight disinfection on high touch surfaces [17]. For a single occupancy patient room, the PX-UV disinfection process consists of two 5 min cycles in the main room; one on either side of the bed; and a single 5 min cycle in the restroom (if applicable). Additive disinfection with PX-UV was not limited to only *C. difficile* isolation rooms. All patient rooms were considered eligible for PX-UV disinfection at patient discharge or transfer.

Utilization of the PX-UV device was assessed using three different metrics; total number of rooms disinfected, total number of PX-UV cycles (2–3 cycles per room), and compliance to protocol. A room was counted as “disinfected” if the appropriate number of cycles were completed, and the appropriate run time was used for each cycle (typically 5 min). The number of cycles and necessary run times are identified for each room within a hospital prior to deployment of the PX-UV device, and this information is stored in the cloud-based portal. The portal software cross-references the pre-determined disinfection requirements with the actual cycles and run time performed to determine whether a room was disinfected correctly. Compliance to protocol was calculated by the total number of rooms disinfected correctly and dividing by the total number of discharges and transfers reported for the month for each unit. This ratio provided a percent compliance, as all targeted unit discharges and transfers were considered eligible for PX-UV disinfection.

### Analysis

Descriptive statistics of the included facilities and aggregate HO-CDI data were compiled. (See Tables 1 and 2) Data was aggregated to facility-level for the negative binomial regressions (n = 1697). After aggregation, the primary purpose was to model HO-CDI count data as a rate by utilizing patient days as an offset time variable within the negative binomial regression model. An offset variable represents the size, exposure, or measurement time, or population size of each observational unit. The regression coefficient for an offset variable is constrained to be 1, thus allowing the model to represent rates rather than counts. Utilizing this method, the PX-UV utilization metrics were compared with HO-CDI rate per 10,000 patient days. Negative binomial regression assumptions were examined using residual diagnostics for hierarchical regression models to analyze the quantile–quantile plot and the expected vs. predicted plots. No significant errors were found.

**Table 1:** Facility demographics (N = 48 hospitals)

Characteristic	N (%)
Region	
Northeast	9 (19%)
Midwest	11 (23%)
South	22 (46%)
West	6 (12%)
Staffed beds	
< 100	5 (10%)
100–299	27 (56%)
300–499	11 (23%)
> 500	5 (10%)
Facility location	
Urban	32 (66%)
Rural	16 (34%)

Northeast (CT, MA, ME, NH, NJ, NY, PA, RI, VT); Midwest (IA, IL, IN, KS, MI, MN, MO, NE, ND, OH, SD, WI); South (AL, AR, DC, DE, FL, GA, KY, LA, MD, MS, NC, OK, SC, TN, TX, VA, WV); West (AK, AZ, CA, CO, HI, ID, MT, NM, NV, OR, UT, WA, WY)

**Table 2:** Descriptive statistics on target units (N = 48 hospitals)

Year	2015	2016	2017	2018	2019
HO-CDI count	587	1054	894	398	150
HO-CDI rate	11.12	9.45	7.63	5.45	3.55
Patient days	525,104	1,115,237	1,172,322	729,967	422,638
PX-UV cycles	33,835	95,115	255,501	252,847	205,342
Rooms disinfected	15,458	39,403	106,692	101,501	71,457
Compliance rate	13.52	17.28	39.78	55.97	64.84
Discharges and transfers	114,354	228,035	268,180	181,339	110,206
No. of observations per year	243	433	503	338	180

*HO-CDI* Hospital Onset *C. difficile*, *PX-UV* Pulsed Xenon Ultraviolet

k-Nearest Neighbor (kNN) machine learning algorithm was utilized to answer the classification question, which was to predict the presence or absence of infection on target units. This analysis compared PX-UV cycles and compliance to protocol to see if there was any relationship between high utilization or compliance and lack of HO-CDI presence. The value of K that was chosen for the model was 10. However, it is worth noting that the model performed well with all values of K. This data remained at the targeted unit level as opposed to facility level (n = 6327). The data was normalized before running the algorithm in order to comply with the standards of machine learning practice. The purpose of this analysis is to portray the relationship between high utilization of the PX-UV device and fewer predicted incidence of HO-CDI, demonstrating the presence of a dose response relationship in a visualization. The algorithm predicts that months with higher utilization of the PX-UV device are less likely to have an occurrence of HO-CDI.

## Results

**Fig. 1:**
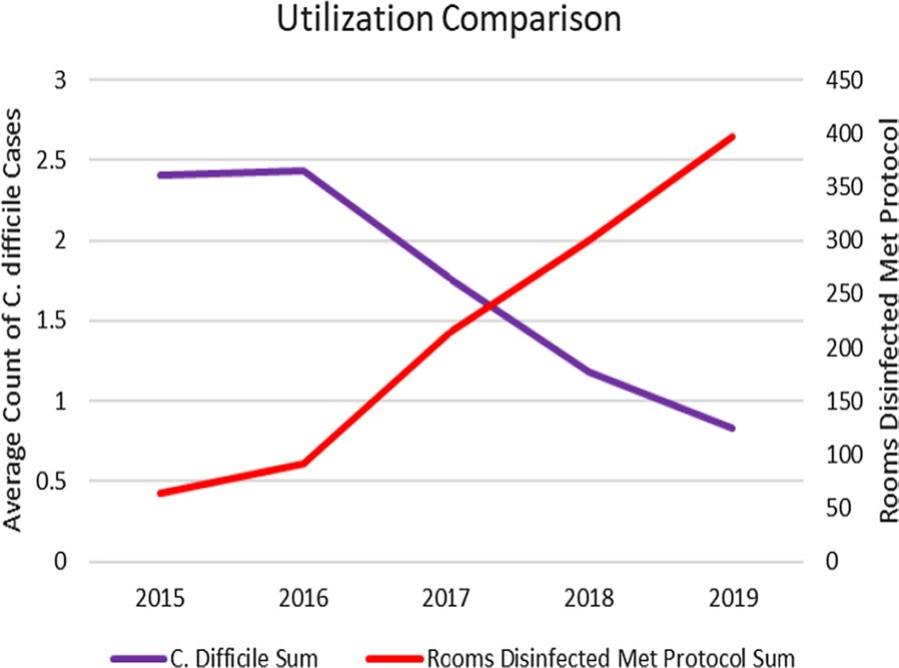
Hospital onset *C. difficile* infection count vs. rooms disinfected by PX-UV

Table 1 shows the descriptions of the facilities included. On average, the facilities were around 300 beds, located primarily in the South region. Table 2 shows the changes in the patient days, discharges and transfers, PX-UV system utilization, and HO-CDI rates and count across the years included in the analysis. From 2015 to 2019, the HO-CDI rate decreased from 11.12 to 3.55 CDI per 10,000 patient days, while PX-UV utilization increased from 33,835 to 205,342 cycles annually. Fig. 1 depicts the trend over time comparing monthly average HO-CDI counts and rooms disinfected. As intervention utilization increases, HO-CDI infection counts decline.

**Fig. 2:**
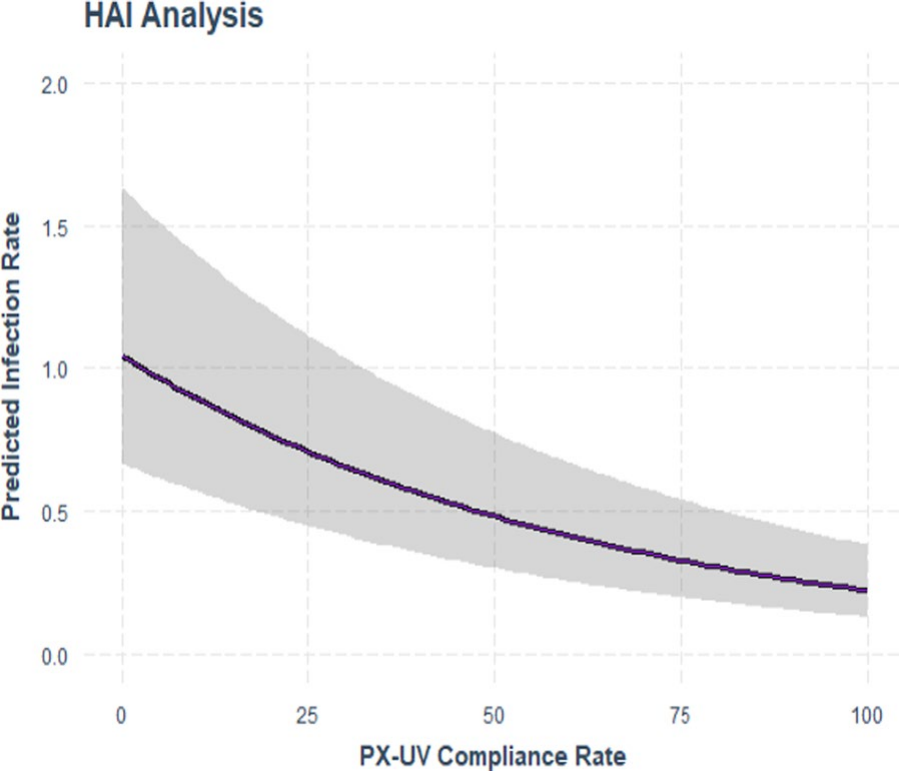
Negative binomial regression prediction plot of PX-UV compliance to protocol and hospital onset *C. difficile* infection rate

Table 3 shows the impact of compliance to protocol on the HO-CDI rate while controlling for the facility in order to prevent any facility from having undue leverage on the result using a negative binomial model with offset for patient days. The model was statistically significant with a p-value of < 0.001. Figure 2 shows with zero compliance (no PX-UV utilization), the monthly infection rate average would be 0.98 per 10,000 patient days across all facilities. At 25% compliance, the model predicts an infection rate of 0.67 per 10,000 patient days, or approximately a 31% infection reduction. At 50% compliance, the model predicts an infection rate of 0.45 per 10,000 patient days, or approximately a 54% infection rate reduction. At 75% compliance, this model predicts an infection rate of 0.31 per 10,000 patient days, which is an infection rate reduction of 68%. Finally, at 100% compliance, the model predicts an infection rate of 0.21 per 10,000 patient days, which is a 78% infection rate reduction. Variance explained in this model is approximately 45%, indicating that other factors (e.g. hand hygiene, manual cleaning, antimicrobial stewardship, etc.) outside of compliance rate and patient days may be key components in mitigation of infection risk. This model best demonstrates the dose–response relationship between increased utilization of the PX-UV device and decreases in HO-CDI rates. When increasing the compliance with disinfection of all discharges and transfers on targeted units, the infection rates on those units decreased.

**T﻿able 3:** Negative binomial regression of PX-UV compliance to protocol & hospital onset *C. difficile* infection rate with facility name as *a control*

	Coef	s.e	95% CI	p-value
PX-UV compliance	− 0.01536	0.001561	− 0.0184, − 0.0123	< 0.001
Intercept	− 0.203			

*PX-UV* Pulsed Xenon Ultraviolet

**Fig. 3:**
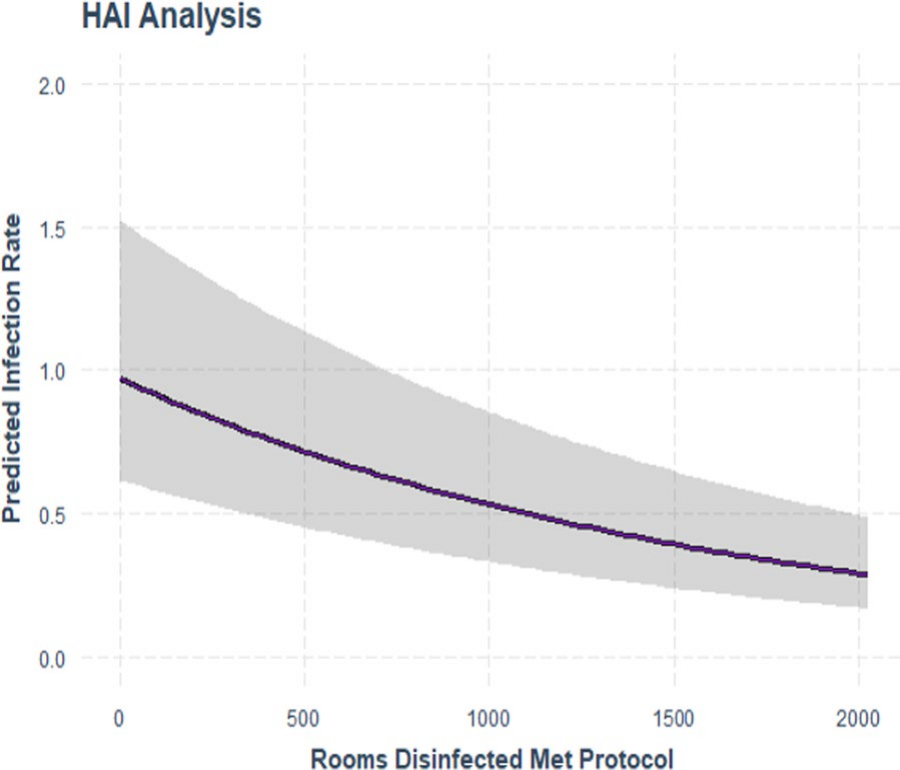
Negative binomial regression prediction plot of rooms disinfected and hospital onset *C. difficile* infection rate

Table 4 shows the impact of the total number of rooms disinfected on the HO-CDI rate while controlling for the facility in order to prevent any facility from having undue leverage on the result using a negative binomial model with offset for patient days. The model was statistically significant with a p-value of < 0.001. The maximum predicted infection rate reduction specific to number of rooms disinfected to protocol as a predictor was 71% assuming 2,026 rooms disinfected. Variance explained in this model was 45%. Figure 3 shows that with an increase in the number of rooms disinfected to protocol, there is a decrease in the number of reported HO-CDI.

**Table 4:** Negative binomial regression of rooms disinfected & hospital onset *C. difficile* infection rate with facility name as a control

	Coef	s.e	95% CI	p-value
Rooms disinfected met protocol	− 0.000597	0.000067	− 0.00073, − 0.000466	< 0.001
Intercept	− 0.3763			

*PX-UV* Pulsed Xenon Ultraviolet

**Fig. 4:**
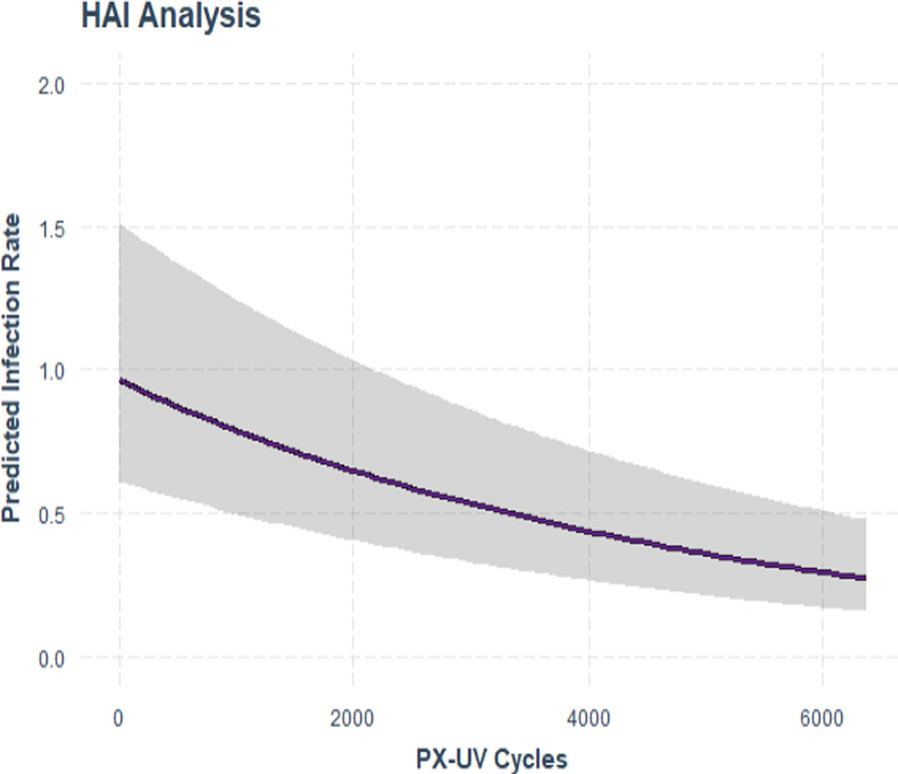
Negative binomial regression prediction plot of PX-UV cycles and hospital onset *C. difficile* infection rate

Table 5 shows the impact of the total number of disinfection cycles on the HO-CDI rate while controlling for the facility in order to prevent any facility from having undue leverage on the result using a negative binomial model with offset for patient days. The model was statistically significant with a p-value of < 0.001. The maximum predicted infection rate reduction specific to PX-UV disinfection cycles as a predictor was 72% assuming 6,370 cycles. Variance explained in this model was similar at 45%. As with the previous analysis, Fig. 4 shows that with an increase in the number of PX-UV disinfection cycles, there is a decrease in the number of reported HO-CDI.

**Table 5:** Negative binomial regression of PX-UV cycles & hospital onset *C. difficile* infection rate with facility name as a control

	Coef	s.e	95% CI	p-value
PX-UV cycles	− 0.0001962	0.000002	− 0.00024, − 0.0001	< 0.001
Intercept	− 0.3928			

*PX-UV* Pulsed Xenon Ultraviolet

**Fig. 5:**
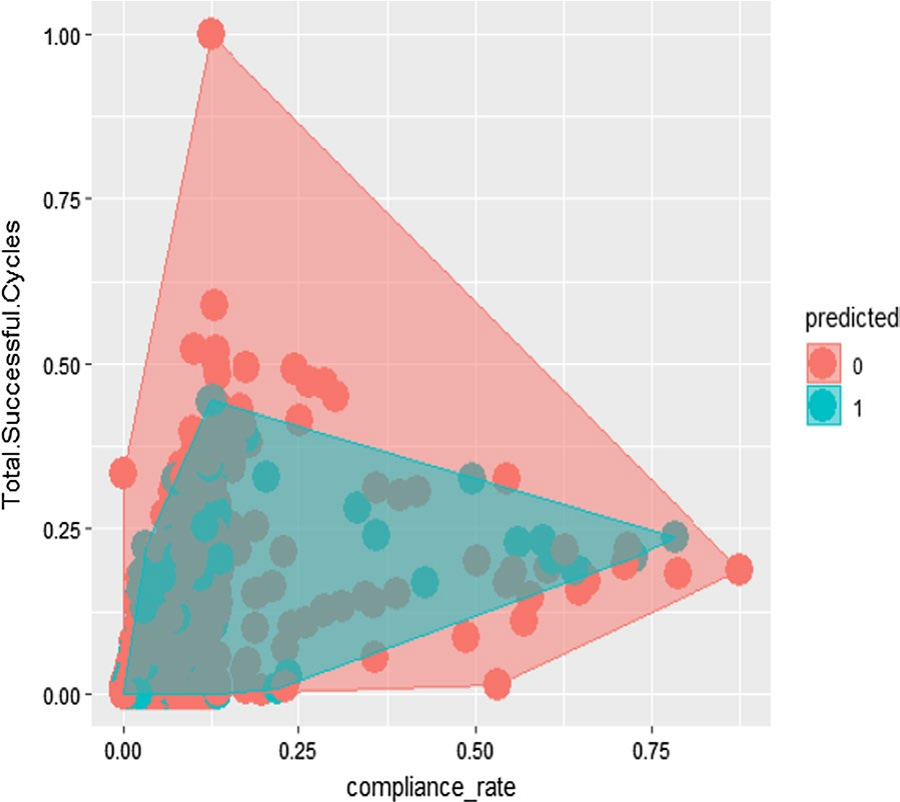
K-nearest neighbor (KNN) model visualization of PX-UV disinfection cycles, compliance rate, and presence of infection

Figure 5 shows the KNN model depicting a lack of HO-CDI cases at higher PX-UV compliance to protocol levels as well as at higher numbers of PX-UV cycles. The data points for months where HO-CDI is present are more closely aggregated in the portion of the chart where total rooms disinfected and overall compliance are the lowest. However, the data points for months where there were no HO-CDI cases are more distributed across areas of higher total usage and compliance. These clustering patterns depict an inverse relationship between presence of HO-CDI and increased utilization of the PX-UV system, and supports the findings of the negative binomial regression analyses.

## Discussion

The aggregate analysis of 48 hospital’s use of the PX-UV system over a 5 year period shows a strong correlation between increased utilization of PX-UV disinfection and decreases in HO-CDI rates. Negative binomial regression models assessing the number of rooms disinfected to protocol, PX-UV disinfection cycles, and the compliance to disinfection protocol showed a statistically significant inverse association with infection rates as PX-UV device use increased. This is consistent with most prior publications on the use of PX-UV systems limited to individual facilities. Instances of PX-UV programs having limited or no impact on HO-CDI rates have been reported, and the authors of these articles state that these results may be attributed to relatively low utilization of the PX-UV device or changes in infection control practices (testing methods, antimicrobial stewardship, hand hygiene etc.) The analyzed dataset is large and robust and represents the aggregate experience of numerous hospitals. The results reflect a statistically significant trend in the decrease of HO-CDI over time associated with an increase in PX-UV system compliance across multiple facilities. These findings increase the likelihood of generalizability of the results to other facilities implementing similar PX-UV programs.

Based on the initial HO-CDI infection rate of 11.12 per 10,000 patient days, 470 infections would have been projected for the 2019 calendar year. Only 150 infections were reported from the included facilities, 320 fewer than projected. A recent meta-analysis showed that the attributable cost of a HO-CDI is $34,149, with an additional attributable length of stay of 7.8 days [9]. Using these estimates, the financial value of this reduced infection risk is approximately 10.9 million USD, and almost 2,500 bed days were made available. A review of studies reporting on 30-day attributable mortality of *C. difficile* infection showed that rates ranged from 5.7 to 6.9% [18]. Using the more conservative figure, an estimated 18 deaths would have been expected to occur among the reduced infections.

The study does not account for other potential changes in infection control (e.g. hand hygiene practices, manual cleaning, antimicrobial stewardship, laboratory testing protocols, etc.), patient risk factors, diagnostics, and/or screening of HO-CDI. The pseudo-R^2^ for the regression models showed that the variance in use of the PX-UV system accounted for approximately 45% of the change in infection rates. This is consistent with the epidemiological understanding of the multi-factorial transmission pathways for HO-CDI. Publications on the trends of HO-CDI rates over long periods of time report that factors such as changes in antimicrobial stewardship practices, *C. difficile* testing practices, and pay-for-performance programs may be important drivers of infection rates [19]. By including a large subset of data where there was no utilization of the PX-UV device, result biases from these temporal changes from other factors may be reduced. Additionally, there is potential selection bias within the customer base to those facilities that had improved or inferior outcomes. The phenomenon of publication bias, wherein results that demonstrate an outcome (either positive or negative) are disseminated whereas results showing no outcome are ignored, may be at play in this data set. Facilities that did not experience changes in their infection rates may not have been motivated to report infection data for analysis. It is important to note not all facilities within this assessment were top tier programs following best practices. There were accounts that did not adopt best practice for implementing PX-UV disinfection, and therefore, did not see similar positive outcomes. Another limitation was the number of hospitals reporting data varied by year, leading to fluctuations in the total number of data points for different time periods in the analysis. This is due to differences in the amount of data reported by each facility over the study time period, leading to differences in each facility’s input in the statistical model. Facilities that reported more HO-CDI and discharge and transfer data could be more influential in the analysis. Finally, missing data for discharges and transfers was populated using predictive mean matching. The analysis would be more robust if discharge and transfer data had been 100% available.

The findings from this study support the use of enhanced disinfection for all patient rooms in healthcare facilities. Multiple models comparing the degree of utilization of a PX-UV disinfection system found an inverse relationship between increased utilization and HO-CDI rates. Each negative binomial regression model reflected similar results whether considering PX-UV cycles, rooms disinfected, or compliance as the predictor. Furthermore, the KNN model suggests that increased utilization of a PX-UV device is a reliable indicator of presence or absence of HO-CDI. Enhanced disinfection system programs require a strategic approach where the facility is meeting consistent compliance goals, which includes completing PX-UV disinfection cycles according to best practices and meeting daily patient demand to ensure opportunities for disinfection are not missed. Enhanced disinfection systems should be considered as part of a comprehensive approach to infection control programs.

## Conclusion

These analyses demonstrate that increased use as measured by frequency of disinfection and compliance to protocol with a PX-UV disinfection program is associated with a decrease in HO-CDI rates. This information can be used by hospitals that are evaluating enhanced disinfection technology to determine best practices.

## Data Availability

The datasets used and analyzed during the current study are available from the corresponding author on reasonable request. The data set was not obtained from a publicly available source.

## Abbreviations

PX-UV: Pulsed Xenon Ultraviolet
KNN: K-Nearest Neighbor
HAI: Hospital Acquired Infection
HO-CDI: Hospital-Onset *Clostridioides difficile* Infection
NHSN: National Healthcare Safety Network
